# Bibliometric and visual analysis of immunisation associated with acute kidney injury from 2003 to 2023

**DOI:** 10.3389/fphar.2024.1388527

**Published:** 2024-07-01

**Authors:** Ling Chen, Jing Hu, Jianrao Lu, Xuezhong Gong

**Affiliations:** ^1^ Department of Nephrology, Seventh People’s Hospital of Shanghai University of Traditional Chinese Medicine, Shanghai, China; ^2^ Department of Nephrology, Shanghai Municipal Hospital of Traditional Chinese Medicine, Shanghai University of Traditional Chinese Medicine, Shanghai, China

**Keywords:** advanced bibliometric analysis, acute kidney injury, immunisation, Web of Science Core Collection, CiteSpace, VOSviewer

## Abstract

**Objective:**

This study aims to conduct a detailed bibliometric and visual analysis of acute kidney injury (AKI) and immune-related research conducted over the past two decades, focusing on identifying emerging trends and key areas of interest.

**Methods:**

The Web of Science Core Collection (WoSCC) was utilised for the meticulous examination of various parameters including publication volume, authorship, geographic distribution, institutional contributions, journal sources, prevalent keywords and citation frequencies. Data were intricately visualised and interpreted using VOSviewer, CiteSpace and Excel 365 software.

**Results:**

Analysis of the WoSCC database revealed 3,537 articles on AKI and immunisation, originating from 94 countries and regions, involving 3,552 institutions and authored by 18,243 individuals. Notably, the top five countries contributing to this field were the United States, China, Germany, Italy and the United Kingdom, with the United States leading with 35.76% of total publications. Among the 3,552 contributing institutions, those in the United States were predominant, with Harvard University leading with 134 papers and 3,906 citations. Key journals driving productivity included Frontiers in Immunology, Kidney International, Journal of the American Society of Nephrology and International Journal of Molecular Sciences, with Kidney International being the most cited, followed by Journal of the American Society of Nephrology and New England Journal of Medicine. Prominent authors in the field included Ronco Claudio, Okusa Mark D and Anders, Hans-Joachim. Co-citation clustering and timeline analysis highlighted recent research foci such as COVID-19, immune checkpoint inhibitors, regulated necrosis, cirrhosis and AKI. Keyword analysis identified “inflammation,” “ischaemia-reperfusion injury,” “sepsis,” “covid-19,” and “oxidative stress” as prevalent terms.

**Conclusion:**

This study provides the first bibliometric analysis of AKI and immune research, offering a comprehensive overview of research hotspots and evolving trends within the field.

## 1 Introduction

Acute kidney injury (AKI), characterised by a sudden and rapid decline in renal function, stems from various physiological and pathological factors (Eisenstein, 2023; Zarbock et al., 2023; Zhao et al., 2023). Moreover, patients with AKI face a potential risk of developing chronic kidney disease (CKD) (Kurata and Nangaku, 2023). Despite considerable advancements in the detection and treatment of AKI, along with research into its pathophysiological mechanisms, the morbidity and mortality rates of AKI continue to rise (Fortrie et al., 2019; Joannidis et al., 2023; Kashani et al., 2023; Zhong et al., 2023). Immunopathophysiological responses can lead to disturbances in both macrocirculatory and microcirculatory functions in the kidney, resulting in functional impairment (Okusa et al., 2017; Inoue et al., 2019). Simultaneous activation of innate immunity components drives kidney inflammation, glomerular and tubular damage and blood–urine barrier disruption (Messerer et al., 2021). Previous studies (Gumbert et al., 2020; Sprangers et al., 2022; Sanz et al., 2023) have demonstrated the critical role of the immune microenvironment and immune response resulting in AKI, offering a promising avenue for novel therapeutic research. Consequently, there is a growing interest in AKI induced by immune dysfunction.

Bibliometric analysis, a scientific knowledge system employed to elucidate further development trends and guide research directions (Liu W.-C. et al., 2023; Xu et al., 2023; Tao et al., 2024), has been utilised in previous studies to explore topics such as ferroptosis (Liu C. et al., 2023), kidney repair (Li and Gong, 2023), global biomarkers trends (Fan and Xu, 2023) and the intellectual base along with global trends (Wang et al., 2023) in AKI research. However, none of these studies have specifically delved into immunisation associated with AKI, an area that has exhibited significant research direction recently. Therefore, this study aims to a comprehensive bibliometric analysis in the field of immunisation associated with AKI, thereby elucidating the hotspots and frontiers of potential researches concerning this area.

## 2 Materials and methods

### 2.1 Data acquisition and search protocol

The Web of Science Core Collection (WoSCC) database was selected for its superior accuracy in document type annotation, making it the optimal choice for literature analysis. A search was conducted on 10^th^ December 2023, within WoSCC, for articles related to the use of immunisation in AKI between 1^st^ January 2004 and 10^th^ December 2023. The search query employed the following formula (((((((((((((((((((((((TS = (Acute Kidney Injury)) OR TS = (Acute Kidney Injuries)) OR TS = (Kidney Injuries, Acute)) OR TS = (Kidney Injury, Acute)) OR TS = (Acute Renal Injury)) OR TS = (Acute Renal Injuries)) OR TS = (Renal Injuries, Acute)) OR TS = (Renal Injury, Acute)) OR TS = (Renal Insufficiency, Acute)) OR TS = (Acute Renal Insufficiencies)) OR TS = (Renal Insufficiencies, Acute)) OR TS = (Acute Renal Insufficiency)) OR TS = (Kidney Insufficiency, Acute)) OR TS = (Acute Kidney Insufficiencies)) OR TS = (Kidney Insufficiencies, Acute)) OR TS = (Acute Kidney Insufficiency)) OR TS = (Kidney Failure, Acute)) OR TS = (Acute Kidney Failures)) OR TS = (Kidney Failures, Acute)) OR TS = (Acute Renal Failure)) OR TS = (Acute Renal Failures)) OR TS = (Renal Failures, Acute)) OR TS = (Renal Failure, Acute)) OR TS = (Acute Kidney Failure) AND TS = (immune).

### 2.2 Bibliometric examination and visual depiction

GraphPad prism (version 8.0.2) was utilised to analyse and visualise annual paper counts, national publication trends and percentages. Additionally, CtieSpace (6.2.4R (64-bit) Premium Edition) and VOSviewer (version 1.6.18) were employed for data analysis and visualisation. Created in 2009 by van Eck and Waltman, (2010), VOSviewer (version 1.6.18) is a Java-based free software facilitating the analysis of extensive literature data by presenting it in map format. CiteSpace (6.2.4R) (Synnestvedt et al., 2005), devised by Prof. Chen Chaomei, enables the construction of literature co-citation network maps, aiding in the visualisation of research outcomes within a specific field. It offers insights into knowledge areas, research frontiers trends and future research trajectories.

### 2.3 Inclusion and exclusion parameters

Inclusion criteria for references in this study comprised: 1) Full text of publications related to the use of immunisation associated with AKI; 2) Articles and reviews written in English; 3) Articles published between 1^st^ January 2004 and 10^th^ December 2023. Exclusion criteria were: 1) irrelevance to the utilisation of immunisation associated with AKI; 2) The articles were conference abstracts, news or briefings.

## 3 Results

### 3.1 Evolution of global publications

**FIGURE 1:**
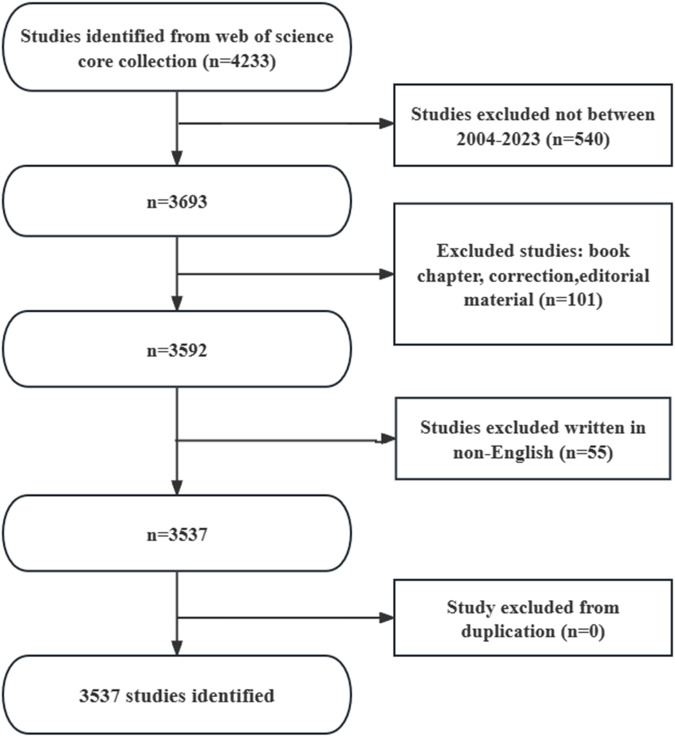
Flow chart of literature screening.

During the period of 1^st^ January 2004 to 10^th^ December 2023, the WoSCC database contained 3,537 articles focusing on the application of AKI and immunisation (Figure 1). Among these, 2,371 articles (67.03%) and 1,166 reviews (32.97%) were identified. The literature encompassed contributions from 94 countries and regions, involving 3,552 institutions and 18,243 authors.

**FIGURE 2:**
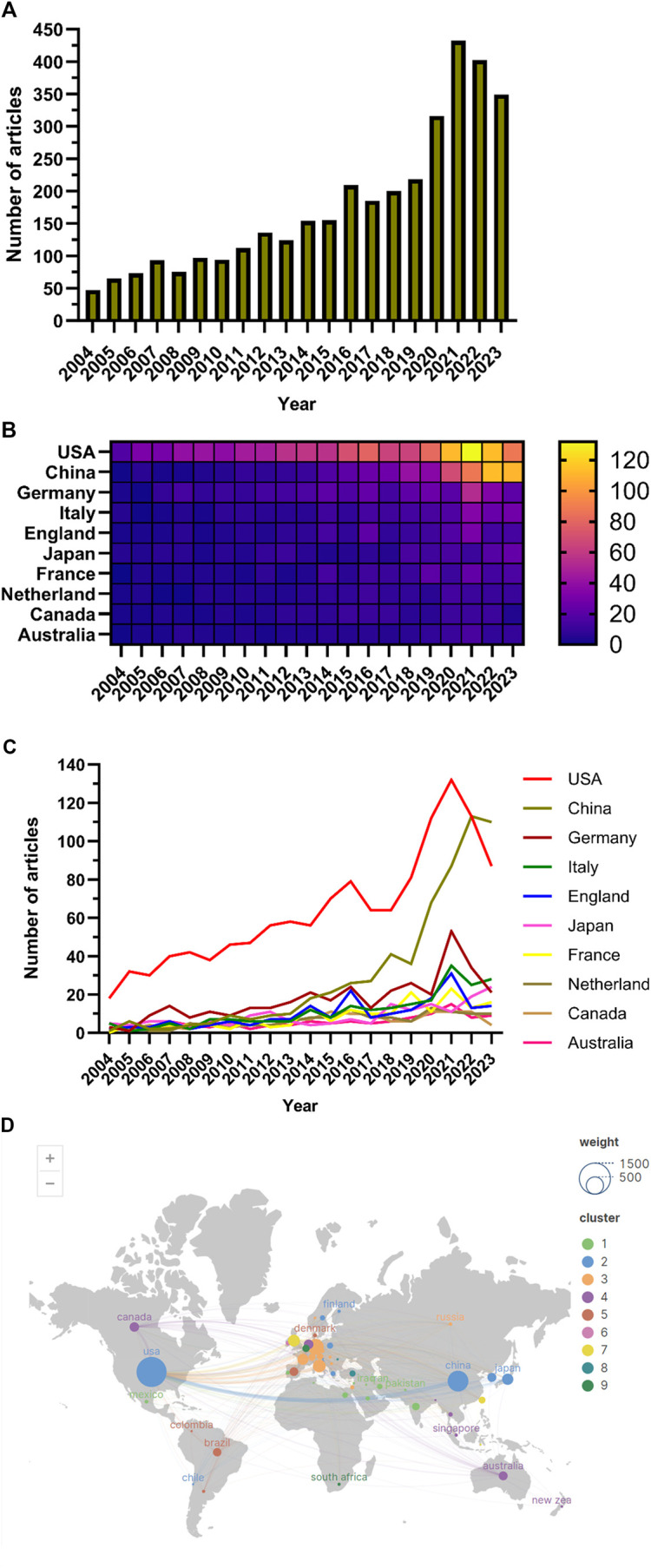
**(A)** Line chart of the number of publications; **(B)** National publication calorimetric map; **(C)**; Line chart of the number of national publications; **(D)** Distribution map of articles published by different countries.

The number of papers published annually exhibited a gradual increase since 2004 (Figure 2A), delineated into three stages. From 2004 to 2010, the growth was modest, with annual publications remaining below 100. Subsequently, from 2011 to 2019, there was a gradual rise in publications. Post-2020, there was a notable surge in publications in this field, reaching a peak in 2021.

### 3.2 International collaborations and contributions

**FIGURE 3:**
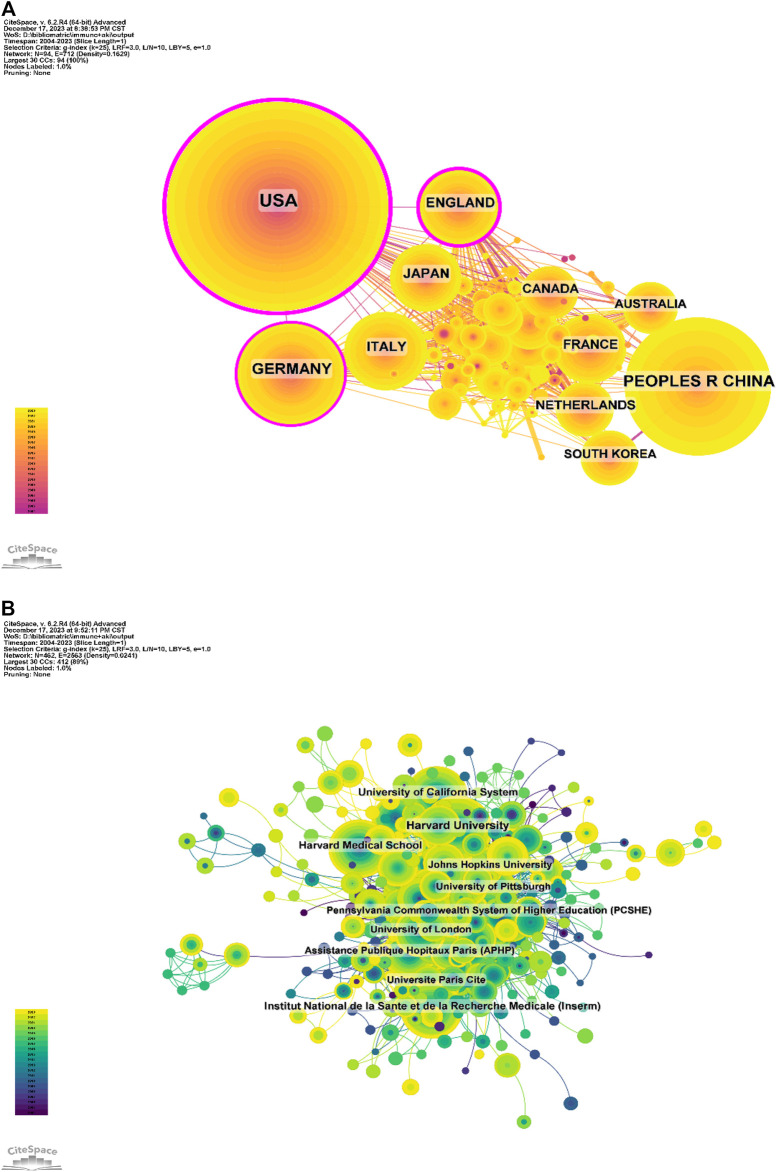
**(A)** Network of national cooperation; **(B)** Network of institutions’ cooperation.

Studies on the application of immunisation in AKI spanned 94 countries and regions (Figures 2B,C,D). Figure 3A illustrates the annual publication volume in the top 10 countries over the past decade, with the United States, China, Germany, Italy and the United Kingdom emerging as the leading contributors. The United States accounted for 35.76% of the total published papers, significantly surpassing other countries. The United States garnered 66,444 citations (Table 1), surpassing other countries and ranking fifth in the citations/publications ratio (52.52%). China ranked second in published papers (603) and number of citations (16,477), with the lowest citation/publication ratio (14.89), suggestive of varying publication quality. The United States demonstrated close collaboration with the United Kingdom, Japan, Italy and Germany, while China exhibited closer collaboration with France, the Netherlands, South Korea and Australia. With high publication numbers, citation frequencies and centrality (0.40), the United States emerged as a pivotal contributor.

**TABLE 1:** National publication scale.

Rank	Country/Region	Article counts	Centrality	Percentage (%)	Citation	Citation per publication
1	United States of America	1,265	0.40	35.76	66,444	52.52
2	China	603	0.06	17.05	16,477	27.33
3	Germany	348	0.14	9.84	16,441	47.24
4	Italy	226	0.07	6.39	9,268	41.01
5	England	194	0.23	5.48	10,069	51.90
6	Japan	177	0.09	5.00	5,097	28.80
7	France	171	0.07	4.83	9,844	57.57
8	Netherlands	130	0.09	3.68	8,074	62.11
9	Canada	126	0.02	3.56	7,180	56.98
10	Australia	112	0.08	3.17	6,741	60.19

### 3.3 Institutional research dynamics

A total of 3,552 institutions contributed to publications in this field. Among the top 10 publications, six from the United States, 3 from France and 1 from the United Kingdom (Table 2; Figure 3B). Harvard University led with the most publications (134 papers, 3,906 citations, 29.15 citations per paper), followed by Harvard Medical School (95 papers, 2,937 citations, 30.92 citations per paper) and Institut National de la Sante et de la Recherche Medicale (Inserm) (92 papers, 1,549 citations, 16.84 citations per paper).

**TABLE 2:** Institutional publication scale.

Rank	Institution	Country	Number of studies	Total citations	Average citation
1	Harvard University	United States of America	134	3,906	29.15
2	Harvard Medical School	United States of America	95	2,937	30.92
3	Institut National de la Sante et de la Recherche Medical (Inserm)	France	92	1,549	16.84
4	University of California System	United States of America	91	1,592	17.49
5	Pennsylvania Commonwealth System of Higher Education (PCSHE)	United States of America	86	1,246	14.49
6	Assistance Publique Hopitaux Paris (APHP)	France	76	1,218	16.03
7	University of Pittsburgh	United States of America	68	5,084	74.76
8	Universite Paris Cite	France	67	2,444	36.48
9	University of London	England	65	1,822	28.03
10	Johns Hopkins University	United States of America	65	4,045	62.23

### 3.4 Enhanced analysis of journals

**FIGURE 4:**
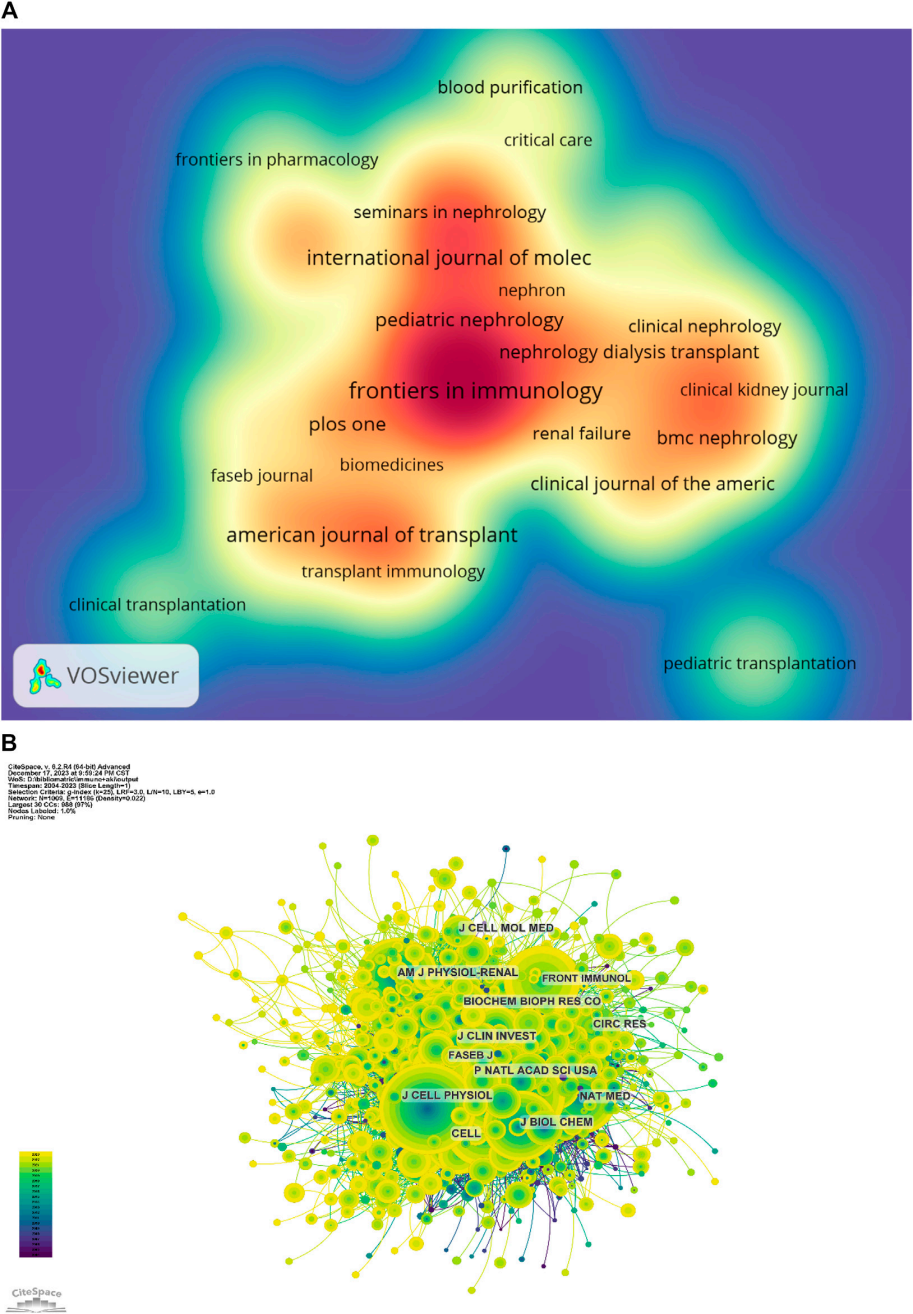
**(A)** Journal publication density chart; **(B)** Journal co-citation network diagram.

Table 3 and Figure 4A present the top 10 most prolific and cited journals. Frontiers in Immunology (133 articles, 3.76%) led in publications, followed by Kidney International (83 articles, 2.35%), Journal of the American Society of Nephrology (78 articles, 2.21%) and International Journal of Molecular Sciences (69 articles, 1.95%). Among these journals, Kidney International boasted the highest impact factor (IF) of 19.6. Furthermore, 90% of these journals were classified as either Q1 or Q2.

**TABLE 3:** Journal publication scale.

Rank	Journal	Article counts	Percentage (3,537)	If	Quartile in category
1	Frontiers In Immunology	133	3.76	7.3	Q1
2	Kidney International	83	2.35	19.6	Q1
3	Journal of the American Society of Nephrology	78	2.21	13.6	Q1
4	International Journal of Molecular Sciences	69	1.95	5.6	Q1
5	American Journal of Transplantation	64	1.81	8.7	Q1
6	Plos One	61	1.72	3.7	Q2
7	American Journal of Physiology-Renal Physiology	56	1.58	4.2	Q1
8	Transplantation	47	1.33	6.2	Q2
9	Paediatric Nephrology	46	1.30	3.0	Q2
10	BMC nephrology	45	1.27	2.3	Q3

Journal influence is determined by co-citations, reflecting its impact on the scientific community. As indicated in Table 4 and Figure 4B, KIDNEY INT (2,205 co-citations) ranked highest in co-citations, followed by J AM SOC NEPHROL (2,017) and NEW ENGL J MED (1,781). Among the top 10 journals, NEW ENGL J MED garnered the highest IF (158.5). All top co-cited journals were either Q1 or Q2.

**TABLE 4:** Journal’s total citations.

Rank	Cited journal	Co-citation	If (2022)	Quartile in category
1	Kidney International	2,205	19.6	Q1
2	Journal of the American Society of Nephrology	2017	13.6	Q1
3	New England Journal of Medicine	1,781	158.5	Q1
4	The Journal of Clinical Investigation	1,457	15.9	Q1
5	Journal of Immunology	1,449	4.4	Q2
6	Nephrology Dialysis Transplantation	1,433	6.1	Q1
7	Plos One	1,351	3.7	Q2
8	Lancet	1,338	168.9	Q1
9	Proceedings of the National Academy of Sciences of the United States of America	1,260	11.5	Q1
10	American Journal of Kidney Diseases	1,077	11.1	Q1

**FIGURE 5:**
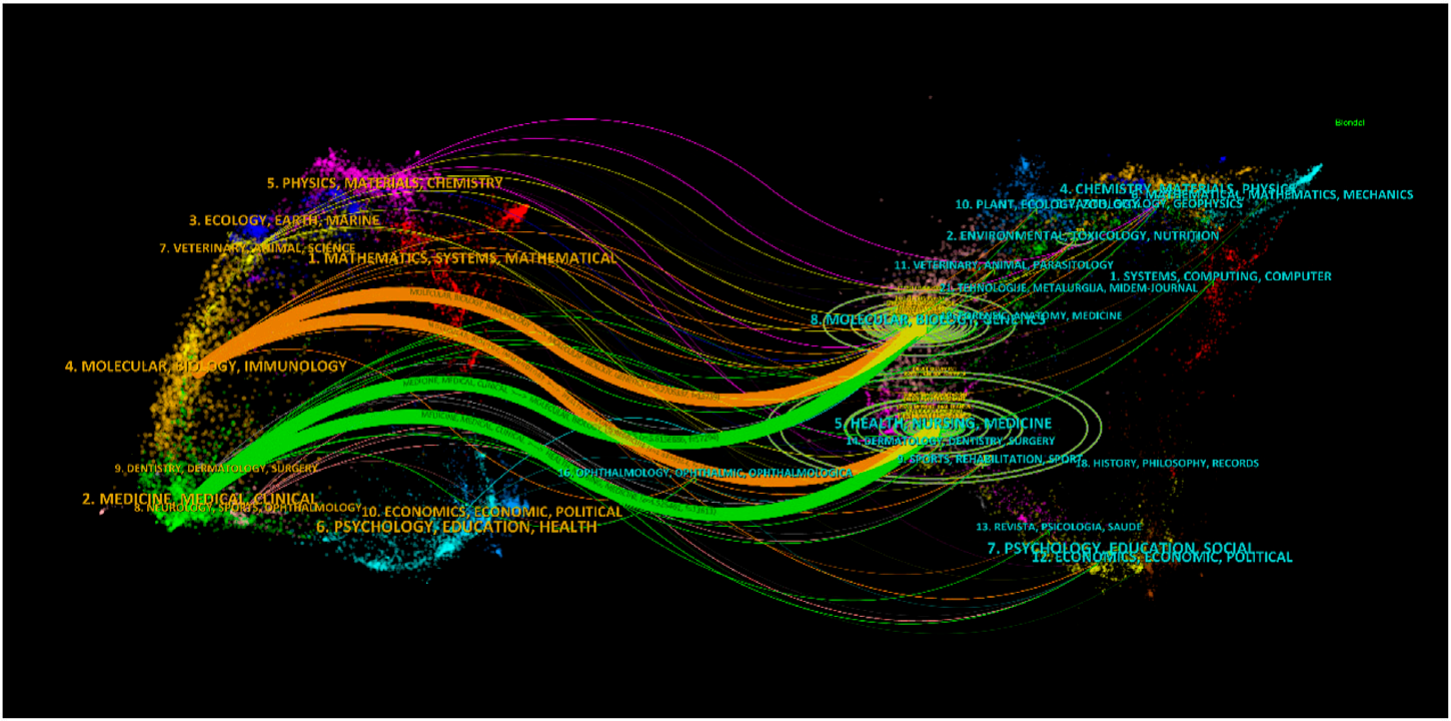
Periodical double overlay diagram.

The subject distribution of academic publications is depicted through dual maps (Figure 5), highlighting citation paths. Four colour citation paths were identified, showcasing research in medicine/medical/clinical fields primarily reported in molecular/biology/genetics and health/nursing/medicine reference research journals. The field of molecular/biology/immunology research was predominately reported in molecular/biology/genetics journals, with cross-referencing to health/nursing/medicine journals.

### 3.5 Analysis of authors and Co-cited authors

**FIGURE 6:**
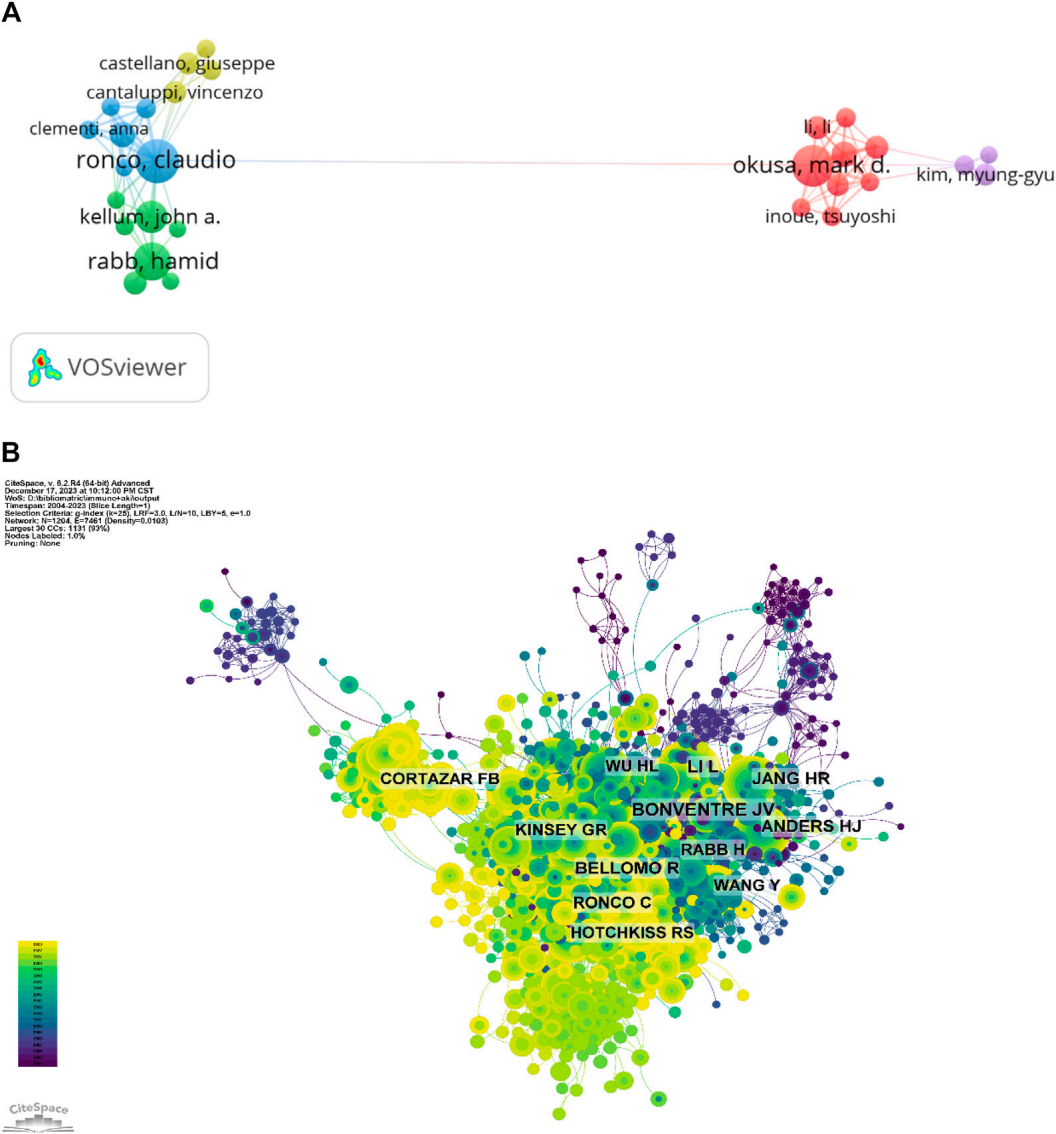
**(A)** Author collaboration network diagram; **(B)** Author co-citation network diagram.

Table 5 and Figure 6A list the 10 authors who have published the most on immunisation associated with AKI. Together, these authors published 208 papers, constituting 5.88% of all papers in the field. Ronco, Claudio emerged as the most prolific author with 32 publications, followed by Okusa, Mark D. (30) and Anders, Hans-Joachim (27). Analysis reveals that five of the top 10 authors are affiliated with institutions in the United States, followed by Italy, Germany, the Netherlands, Brazil and China. Further examination using CiteSpace visualises the network among authors, identifying Anders hans-joachim as the leading author in the field. Table 5 and Figure 6B delineate the top 10 co-cited and most cited authors, respectively. The 56 authors have been cited more than 50 times, indicating significant reputation and influence. Notably, most co-cited authors include LAMBIN P (367 citations), GILLIES RJ (328 citations) and LI H (210 citations).

**TABLE 5:** Publications and co-citations of the top 10 authors.

Rank	Author	Count	Location	Rank	Co-cited author	Citation
1	Ronco, claudio	32	Italy	1	BONVENTRE JV	248
2	Okusa, mark d	30	United States of America	2	LI L	219
3	Anders, hans-joachim	27	Germany	3	ANDERS HJ	183
4	Rabb, hamid	26	United States of America	4	JANG HR	183
5	Kellum, john a	20	United States of America	5	BELLOMO R	176
6	Perazella, mark a	19	United States of America	6	RABB H	171
7	Florquin, sandrine	14	Netherlands	7	RONCO C	162
8	Saraiva camara, niels olsen	14	Brazil	8	KINSEY GR	160
9	Huang, liping	13	China	9	CORTAZAR FB	150
10	Sarwal, minnie m	13	United States of America	10	WU HL	139

### 3.6 Analysis of Co-cited references

**FIGURE 7:**
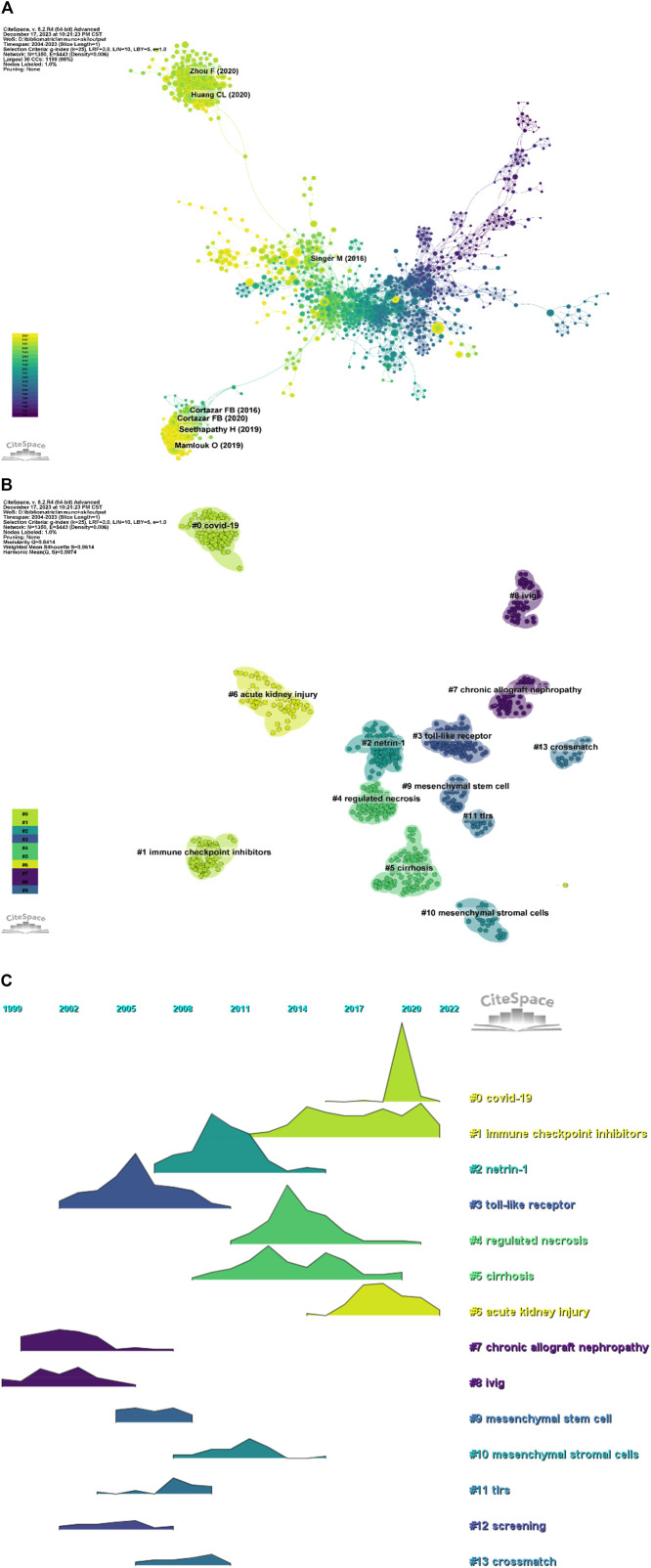
**(A)** Network diagram of co-cited documents; **(B)** Clustering diagram of co-cited documents; **(C)** Volcano map of co-cited references.

In a 1-year time slice spanning 2004 to 2023, the co-citation reference network comprised 1,350 nodes and 5,443 links (Figure 7A). The top 10 most cited articles (Table 6) feature a paper from the Clinical Journal of the American Society of Nephrology (IF = 9.8) entitled ‘The Incidence, Causes, and Risk Factors of Acute Kidney Injury in Patients Receiving Immune Checkpoint Inhibitors’ as the most frequently cited reference. Authored by Seethapathy Harish, the paper discusses the escalating use of immune checkpoint inhibitors (ICIs) in oncology.

**TABLE 6:** Co-citation of top10 literature.

Rank	Title	Journal if (2021)	Author(s)	Total citations
1	The Incidence, Causes, and Risk Factors of Acute Kidney Injury in Patients Receiving Immune Checkpoint Inhibitors	Clinical Journal of the American Society of Nephrology (IF = 9.8)	Seethapathy H	84
2	Clinical Features and Outcomes of Immune Checkpoint Inhibitor-Associated AKI: A Multicenter Study	Journal of the American Society of Nephrology (IF = 13.6)	Cortazar FB	80
3	Clinicopathological features of acute kidney injury associated with immune checkpoint inhibitors	Kidney International (IF = 19.6)	Cortazar FB	78
4	Nephrotoxicity of immune checkpoint inhibitors beyond tubulointerstitial nephritis: single-centre experience	Journal for Immunotherapy of Cancer (IF = 10.9)	Mamlouk O	74
5	The Third International Consensus Definitions for Sepsis and Septic Shock (Sepsis-3)	Jama-Journal of the American Medical Association (IF = 120.7)	Singer M	63
6	Clinical features of patients infected with 2019 novel coronavirus in Wuhan, China	Lancet (IF = 168.9)	Huang CL	62
7	Clinical course and risk factors for mortality of adult inpatients with COVID-19 in Wuhan, China: a retrospective cohort study	Lancet (IF = 168.9)	Zhou F	62
8	Association of Acute Interstitial Nephritis With Programmed Cell Death 1 Inhibitor Therapy in Lung Cancer Patients	American Journal of Kidney Diseases (IF = 13.2)	Shirali AC	60
9	Renal histopathological analysis of 26 *postmortem* findings of patients with COVID-19 in China	Kidney International (IF = 19.6)	Su H	59
10	Immune-Related Adverse Events Associated with Immune Checkpoint Blockade	New England Journal of Medicine (IF = 158.5)	Postow MA	55

Co-cited reference clustering and temporal clustering analysis (Figures 7B, C) reveal various research hotspots over time, such as toll-like receptors (cluster 3), chronic allograft nephropathy (cluster 7) and ivig (cluster 8) in earlier periods, progressing to Netrin-1 (cluster 2), mesenchymal stem cell (cluster 9) and mesenchymal stromal cells (cluster 10), tlrs (cluster 11), screening (cluster 12), crossmath (cluster 13) as mid-term research hotspots. Currently, COVID-19 (cluster 0), immune checkpoint inhibitors (cluster 1), regulated necrosis (cluster 4), cirrhosis (cluster 5) and acute necrosis (kidney) injury (cluster 6) are the research trends in this field.

### 3.7 Keyword analysis

**FIGURE 8:**
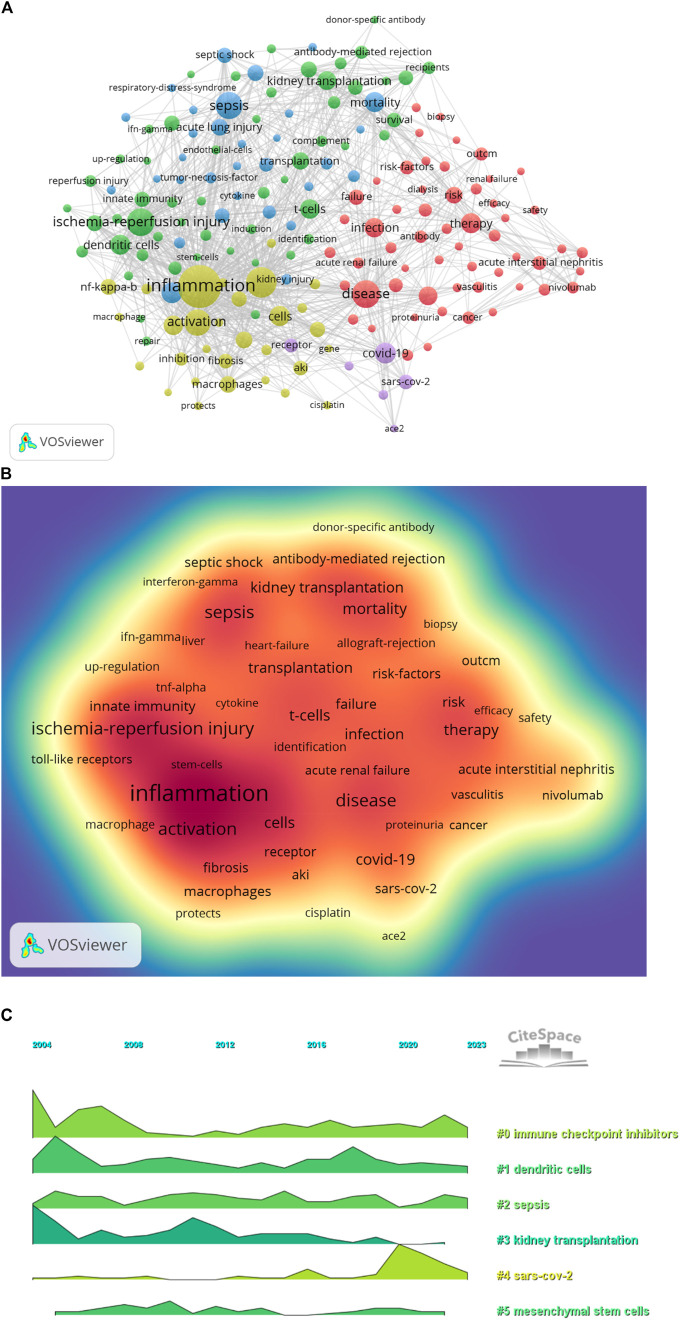
**(A)** Keyword clustering network diagram; **(B)** Keyword density map; **(C)** Keyword volcano map.

Keyword co-occurrence analysis in VOSwiever highlights inflammation (575) as the most popular keyword, followed by expression (330), ischaemia-reperfusion injury (283), disease (279) and sepsis (267) (Figures 8A,B; Table 7). Filtering out redundant keywords, a network of 176 keywords appearing at least 28 times reveals five distinct clusters. Group 1 (red) encompasses 56 keywords, including disease, glomerulonephritis, therapy, infection, failure, risk, safety, cancer, toxicity, antibody, kidney transplant, biopsy and vasculitis. Group 2 (green) comprises 53 keywords, including acute rejection, complement, tolerance, donor-specific antibody, survival, immunosuppression, recipients, T cell, TGF-β, toll-like receptors and dendritic cells. Group 3 (blue) contains 31 keywords, including sepsis, septic shock, acute lung injury, cytokines, dysfunction, model, neutrophil, rats, liver and necrosis factor-alpha. Group 4 comprises 30 keywords (yellow), including inflammation, activation, macrophages, fibrosis, renal injury, pathway, autophagy, cisplatin, protects, immunity, apoptosis and cell death. Group 5 (purple) contains six keywords, including ace2, coronavirus, covid-19, receptor, responses and sars-cov-2. Additionally, a volcano map generated using CiteSpace visualises the evolution of study hotspots over time (Figure 8C).

**TABLE 7:** High-frequency keywords.

Rank	Keyword	Counts	Rank	Keyword	Counts
1	inflammation	575	11	dendritic cells	164
2	expression	330	12	mortality	162
3	ischaemia-reperfusion injury	283	13	t cells	159
4	disease	279	14	apoptosis	156
5	Sepsis	267	15	infection	153
6	activation	256	16	glomerulonephritis	148
7	covid-19	174	17	kidney transplantation	148
8	oxidative stress	171	18	transplantation	131
9	Cells	167	19	acute lung injury	123
10	therapy	165	20	risk	123

### 3.8 Highlighting Co-cited references and keywords

**FIGURE 9:**
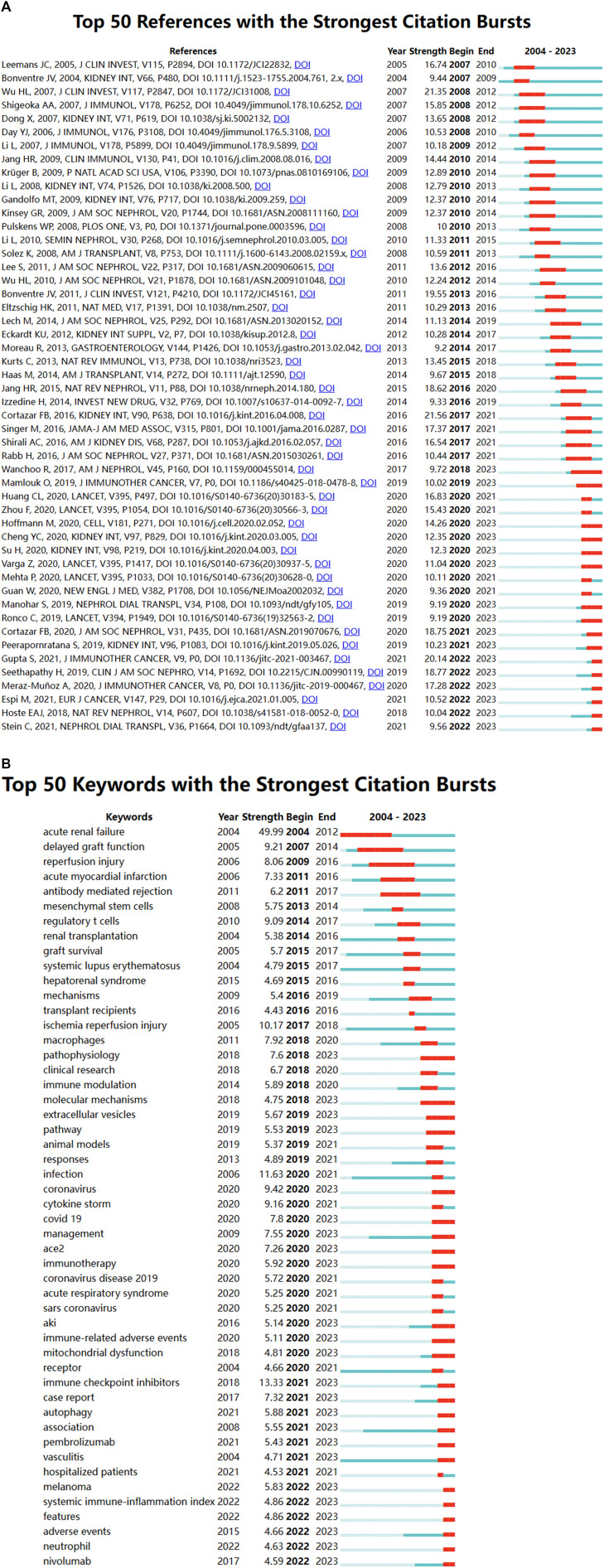
**(A)** Top 50 references with the strongest citation bursts; **(B)** Top 50 keywords with the strongest citation bursts.

CiteSpace analysis, shown in Figure 9A **,** identified the 50 most reliable citation bursts, with the highest citation rate (21.56) attributed to “Clinicopathological features of acute kidney injury associated with immune checkpoint inhibitors” by Frank B. Cortazar. Notably, all 50 references were published between 2004 and 2023, indicating that these papers were frequently cited over nearly 2 decades. Importantly, 16 of these papers are currently experiencing their citation peak, underscoring ongoing interest in immune-related AKI research. Additionally, the analysis of the 257 strongest burst keywords (Figure 9B) highlights current research hotspots in the field and potential future research directions in the field.

## 4 Discussion

This study is the first bibliometric analysis aiming to elucidate the topic of immune researches related to AKI. Our objective was to provide a comprehensive overview of this field during the include peroid using bibliometric software. Through the visualisation of quantitative data conducted, valuable insights into research hotspots and trends were obtained.

### 4.1 Bibliometric information

A search of the WoSCC database from 1^st^ January 2004 to 10^th^ December 2023, yielded 3,537 articles in the field of AKI and immunisation (Figure 1). The literature involved contributions from 94 countries and regions, 3,552 institutions and 18,243 authors. Publications rates showed a gradual increase since 2004 (Figure 2A); however, from 2004 to 2010, the growth declined. From 2011 to 2019, published papers gradually increased, and post-2020, they increased rapidly, peaking in 2021. This suggests a growing interest in this area in recent years.

The top five countries contributing to annual publication volumes were the United States, China, Germany, Italy and the United Kingdom. The United States stood out with the highest citation count (66,444 citations) (Table 1), far surpassing other countries, and ranked fifth among all countries in the citations/publications ratio. China ranked second in published papers and citations but demonstrated a lower citation/publication ratio.

Among the top 10 publications, six were from the United States, three from France and one from the United Kingdom (Table 2; Figure 3B). Harvard University emerged as the leading contributor, followed by Harvard Medical School and Institut National de la Sante et de la Recherche Medicale (Inserm), respectively.

Table 3 and Figure 4A list the top 10 most produced and cited journals. Frontiers in Immunology was revealed as the most published journal in the field, followed by Kidney International, Journal of the American Society of Nephrology and International Journal of Molecular Sciences. Journal influence is determined by how often it is co-cited, which, in turn, indicates its impact on the scientific community. The journal with the most common citations was KIDNEY INT (2,205), followed by J AM SOC NEPHROL (2,017) and NEW ENGL J MED (1,781) (Table 4; Figure 4B **)**. It as well demonstrated top journals on the topic included professional journals of nephrology and other fields like molecular sciences, indicating the multidisciplinary nature of this field.

The top 10 authors published 208 papers or 5.88% of all papers in the field (Table 5; Figure 6A), with Ronco, Claudio leading with the most publications (32), followed by Okusa, Mark D (30) and Anders, hans-joachim (27). Further analysis identified Anders hans-joachim, the third most published and cited author, as a leading figure in the field.

Among the top 10 most cited articles (Table 6), a paper from the Clinical Journal of the American Society of Nephrology (IF = 9.8) entitled ‘The Incidence, Causes, and Risk Factors of Acute Kidney Injury in Patients Receiving Immune Checkpoint Inhibitors’ is the most frequently cited reference. Seethapathy Harish, lead author of the paper, discusses the rapid increase in the use of ICIs in oncology. They intended to figure out the frequency, severity and causes of AKI in populations treated with ICIs. Treated with ICIs, AKI is common in patients with varying causes. Nevertheless, the role of proton pump inhibitors (PPI) and other agents that induce nephritis in persistent AKI required further elucidation.

### 4.2 Hotspots and frontiers

The top 5 keywords were inflammation (575), followed by ischaemia-reperfusion injury (283), sepsis (267), covid-19 (174) and oxidative stress (171) (Figures 8A,B; Table 7). CiteSpace analysis, shown in Figure 9A **,** identified the 50 most reliable citations burst. Among them, the reference with the highest citation rate (21.56) was “Clinicopathological features of acute kidney injury associated with immune checkpoint inhibitors” by Frank B. Cortazar. This article reveals ICIs-induced AKI is a new entity that presents with clinical and histologic features similar to other causes of drug-induced acute tubulointerstitial nephritis, though with a longer latency period. Glucocorticoids appear to be a potentially effective treatment strategy. Hence, AKI due to ICIs may be caused by a unique mechanism of action linked to reprogramming of the immune system, leading to loss of tolerance. All 50 references were published between 2004 and 2023, with 16 currently peaking. These observations suggest that the application of immunity to AKI research will continue to be of interest in the future. Of the 257 strongest burst keywords in the field, we focus on the 50 keywords with the strongest mutations (Figure 9B), which represent current research hotspots and represent possible research directions. It is described and discussed from the following three aspects related mechanisms, associated immune cells and immune responses as well as underlying pathways of action.

#### 4.2.1 Related mechanisms

The immune microenvironment and immune response are crucial to the occurrence and development of AKI, and might be a new therapeutic target research direction (Gumbert et al., 2020; Xu L. et al., 2024). Regulating inflammatory response and enhancing the regenerative potential of stem cells in the inflammatory microenvironment contribute to the regeneration of renal tubular epithelium. Studies have shown (Sprangers et al., 2022; Deng et al., 2023) that the immune response induced by AKI is closely related to the formation of complex cytokine networks by T cell subsets (Th1, Th2, Th17, Th9, Th22, etc.) in renal tubular epithelial tissue, and targeted immunity can inhibit the progression of AKI. Therefore, the exploration of molecules or drugs that can control the inflammatory response, reverse the pro-inflammatory immune microenvironment, and improve the differentiation ability of stem cells to promote the regeneration of renal tubular epithelial tissue is currently a hot spot in the clinical treatment of AKI.

Both inflammation and oxidative stress play a crucial role in the potential molecular mechanisms of acute kidney damage process (He et al., 2017; Choi et al., 2022; Fan et al., 2023). Addressing inflammatory reactions is a viable approach in the treatment of both CKD and AKI. Oxidative stress performs a fundamental function in the advancement of renal disorders and the progression of kidney-related problems. Inflammation and oxidative stress are inextricably related, jointly causing and exacerbating the effects of the other. Chuan Huang Fang (CHF) is a Chinese herbal formulation synthesized by Professor Xuezhong Gong in Shanghai Municipal Hospital of Traditional Chinese Medicine for the treatment of AKI on CKD (A on C) patients (Chen et al., 2022). Previous study (Gong et al., 2020) has demonstrated that after treatment with CHF, serum levels of oxidation-antioxidant related biomarkers malondialdehyde (MDA), superoxide dismutase (SOD) and heme oxygenase-1 (HO-1) in A on C patients showed significant changes compared with those before treatment, which showed levels of HO-1 and SOD in patients with A on C were higher than those before treatment, and MDA levels were lower. Therefore, it is believed that CHF might have certain antioxidant effect on patients with A on C. As an inflammatory signal that has been paid much attention to in recent years, NOD-like receptor protein-3 (NLRP3) inflammasome plays an important role in the occurrence and development of AKI (Li Y. et al., 2024; Xue et al., 2024). Another study (Gong et al., 2021) have shown that CHF has a better inhibitory effect on NLRP3 inflammasome, which might be the effective treatment mechanisms of CHF. The antioxidant effects of CHF could also inhibit the increase of NLRP3 inflammatorome in patients with A on C to further exert the renal protection effects. Tetramethylpyrazine (TMP), an active component in both CHF and the medicinal herbs Ligusticum wallichii (Chuanxiong), has the potential to prevent AKI via a variety of processes, including ameliorating oxidative stress damage, suppressing inflammatory responses, deterring apoptotic cell death of intrinsic renal cells, and modulating autophagy (Li and Gong, 2022).

The pathogenesis of sepsis associated acute kidney injury (S-AKI) is complex. It is mainly related to renal hemodynamic changes, inflammation, oxidative stress injury, ischemia reperfusion injury, apoptosis, coagulation dysfunction, adaptive mechanism of energy metabolism of renal tubular epithelial cells, gene differences and expression, etc (Kaushal and Shah, 2016; Yang et al., 2021; He et al., 2024). Emodin might play a renal protective role in S-AKI mice by enhancing the expression of Nrf2 and AUF1 proteins, regulating related oxidation-reduction enzyme, enhancing antioxidant stress ability, inhibiting inflammatory response (Ding et al., 2023). Studies reveal that (Yancy et al., 2017) the mechanism of CRRT in the treatment of S-AKI is primarily through convection and adsorption of soluble inflammatory mediators and toxins, reducing immune suppression, inhibiting lymphocyte apoptosis, enhancing immune function, inhibiting inflammatory cascade and improving renal function. A study revealed Changpu Yujin Decoction combined with CRRT is more effective than CRRT alone in the treatment of S-AKI, which could be attributed to the effective suppression of inflammation through reduced serum PCT levels, CRP and T cell subset regulations (Long et al., 2020).

The occurrence and development of renal ischemia-reperfusion injury (IRI) might mediate inflammatory response through inflammation-related signaling pathways, and blocking inflammatory response plays a very important role in improving the pathological status of AKI (Li et al., 2022; Creed et al., 2024). Relevant studies (Vande Walle and Lamkanfi, 2024; Xu K. et al., 2024) have shown that pyroptosis is a key mechanism of renal IRI. Pyroptosis is an inflammation-related programmed cell necrosis, following damage to NLRP3. The NLRP3 inflammasome is activated, which in turn promotes the release of inflammatory cytokines interleukin-1β (IL-1β) and IL-18 (Xue et al., 2024). Dl-3-N-butylphthalide (NBP) not only plays an anti-inflammatory role, but also participates in anti-oxidative stress, promoting angiogenesis, and protecting the function of the blood-brain barrier (Han et al., 2021). Studies (Dong et al., 2021; Zhu T. et al., 2024) have shown that NBP could reduce inflammation, prevent inflammatory damage caused by pyroptosis, improve microcirculation, etc. Besides, it has a good preventive effect on IRI in heart, brain, and other tissues. Zhang et al. (2023) revealed NBP may downregulate the activity of NF-κB/NLRP3 signaling pathway and reduce the expression levels of cell pyroptosis-related proteins and inflammatory factors after renal IRI, thereby suppressing cell pyroptosis and alleviating renal IRI.

#### 4.2.2 Associated immune cells and immune responses

The activation of immune cells and subsequent immune response are important factors that contribute to the further deterioration and persistence of renal function post-AKI. Therefore, the study of immune response in AKI is crucial for understanding its pathophysiology and developing novel preventive and therapeutic strategies. Various immune cells, such as neutrophils, dendritic cells (DC), macrophages, natural killer (NK) cells, NKT cells, CD4 + T cells and regulatory T cells (Tregs), are activated after AKI incidence and participate in kidney inflammation, with neutrophils, DC and Tregs playing important roles. Notably, the activation of immune cells and the resulting cascade reactions play an important role in AKI. Accordingly, interventions targeting these cells in animal studies have been demonstrated to reduce or worsen kidney damage. Targeting these cells may offer promising avenues for clinical intervention.

T cells might have pathogenic and reparative effects resulting in AKI, but it is relatively unknown what is the certain mechanisms regulating T-cell responses. Noel et al. (2023) investigated the roles of the novel immune checkpoint molecule T cell immunoreceptor with Ig and immunoreceptor tyrosine-based inhibitory motif domains (TIGIT) in kidney T cells and AKI outcomes. TIGIT expression increased in mouse and kidney T cells, which would lead to worse AKI outcomes. Thus, it might be a potential therapeutic target for AKI. A experimental study (Packialakshmi et al., 2020) have demonstrated innate immune system is a leading cause to AKI (Uchida et al., 2023).

AKI is considered a common complication for patients receiving ICIs treatments, originating from either kidney injury or immune activation resulting in acute interstitial nephritis (AIN). Megan et al. (Baker et al., 2022) reported higher mortality in AKI patients unrelated to ICI than those with AIN as its underlying aetiology. It is reported tubulointerstitial nephritis as the most common renal lesion caused by ICIs (Mamlouk et al., 2019). ICIs are emerging immunotherapy that has revolutionised several kinds of malignancies (Cortazar et al., 2020). By targeting inhibitory receptors expressed on T lymphocytes, other immune cells and tumour cells, these monoclonal antibodies enhance tumour-directed immune responses, ensuring high efficacy in treating a broad spectrum of malignancies (Wei et al., 2018).

Immune-related AKI (irAKI) is the primary complication of all immune-related adverse events (Franzin et al., 2020). Correlations of irAKI incidence and causing factors have been studied, including impaired renal function at baseline, use of a PPI, ipilimumab, extrarenal irAEs, ICIs combined with chemotherapy or autoimmune disease history (Cortazar et al., 2020; Abdelrahim et al., 2021; Seethapathy et al., 2021). However, it is remain unclear all the potential risk factors for irAKI. The outcomes of patients are greatly influenced by ICIs-induced irAKI. Therefore, when irAKI occurs, supportive treatments should be inniated instead of ICIs. There are still a lack of studiess on irAKI caused by ICIs. To our relief, the increasing number of clinical trials involving ICIs enable high-quality researches on irAKI (Liu F. et al., 2023).

The occurrence of AKI induced by ICIs may be related to the pharmacological action of the drugs themselves, leading to the release of immune brakes. Additionally, when ICIs inhibit CTLA-4/PD-1/PD-L1, the “immune brake” of the body is released, which not only strongly activates the immune ability of T cells to tumour cells, but also leads to the decrease in the tolerance of the kidney to endogenous antigens, thus triggering AKI (Izzedine et al., 2019). Furthermore, the occurrence of ICI-induced AKI could be attributed to the “multi-hit” of combined drugs on the kidney. ICIs not only affect the normal immune tolerance of the kidney to endogenous antigens but also reduce the immune tolerance of the body to other combined drugs (Wanchoo et al., 2017). The systemic immune-inflammatory index (SII), a novel inflammatory index based on neutrophils, lymphocytes and platelets, has shown promise in predicting the prognosis of various malignant tumours and inflammatory diseases (Tian et al., 2022; Chen et al., 2023). Recently, researchers have also found that SII could predict acute pancreatitis and contrast-induced AKI (Li and Liu, 2022), further highlighting its potential utility in AKI management.

AIN emerges as the most common biopsy-proven diagnosis in patients on ICI therapy experiencing AKI (Izzedine et al., 2019). The mechanism underlying this phenomenon remains elusive, but it is hypothesised that ICIs may provoke unregulated cell responses and proliferation in the tubulointerstitium. Additionally, it is plausible ICIs result in the loss of immune tolerance and activation of memory T cells previously primed by other haptens causing AIN (Sury et al., 2018). Studies have found a significant proportion of patients (14 of 19, 73%) on ICIs with biopsy-proven AIN had prior exposure to drugs resulting in AIN (Cortazar et al., 2016; Seethapathy et al., 2019).

Immunotherapy-related AIN could result from the loss of tolerance of drug specific effector T cells upon inhibition of PD-1 signalling. They could have experienced nephritogenic drug exposure. Another possible mechanism involves the procedure of autoimmunity to kidney self-antigens following the loss of self-tolerance and potentiation of antigen recognition upon blocking the CTLA-4 or PD-1 pathway, which regulates immunity at peripheral and target organ levels, respectively (Mamlouk et al., 2019). Furthermore, individuals recovering from AKI have great possibility of developing CKD. The transition of underlying mechanisms might involve sustained activation of renal innate immunity, renal inflammation and fibrosis, et al. These existing factors offer a plausible explanation for increase in the transition rate from AKI to CKD (Albino et al., 2021).

#### 4.2.3 Underlying pathways of action

It is gradually recognised mitochondrial dysfunction as a critical factor to AKI. Th of damaged mitochondria mediating AKI are multifactorial and complex. The cyclic GMP-AMP synthase (cGAS) stimulator of the interferon genes (STING) (cGAS-STING) pathway detects cytosolic DNA and induces innate immunity. Studies have shown that mitochondrial DNA (mtDNA) depletion and repletion might result in tubular inflammatory responses via the cGAS-STING signal activation by cytosolic mtDNA. Similarly, Hiroshi et al. (Maekawa et al., 2019) concluded mitochondrial dysfunction and subsequent mtDNA-cGAS-STING pathway activation as critical regulator of AKI. There have been showing great importance of circulating mtDNA and related pathways in the progression of AKI, and regulating the related proteins could serve as an potential strategy to alleviate AKI (Liu et al., 2021). Qi et al. (2023) observed the activation of cGAS-STING pathway in cisplatin induced AKI. After inhibiting this pathway, the activation of TNF-α, IL-6, IL-8, ICAM-1, MCP-1 and other inflammatory factors were inhibited, thus improving the renal tissue structure, and promoting the recovery of renal function. Luo et al. (2022) found in human kidney-2 (HK-2) cultured *in vitro* that the expressions of cGAS and STING were significantly increased after cisplatin intervention, while significantly decreased after β-hydroxybutyrate treatment, and the autophagy, inflammation and apoptosis of cells were also decreased.

Studies (Tang et al., 2018; Pabla and Bajwa, 2022) have revealed that disruption of mitochondrial homeostasis related to mitochondrial biogenesis, mitochondrial autophagy and increased membrane permeability are all related to renal tubule injury and inflammation in AKI. Li et al. (2023) showed that phosphoglycerate mutase 5 (PGAM 5) mediated Bax dephosphorylation induced mtDNA release and cGAS-STING pathway activation are closely related to inflammation and kidney injury. In mice models with PGAM5 or cGAS knockout, renal injury and inflammation caused by IRI were alleviated to varying degrees. Besides, the same results were observed in the renal proximal tubule epithelial cells of mice treated with hypoxia/reoxygenation *in vitro*. Relevant study (Feng et al., 2022) has shown that receptor interacting protein 3 (RIP3) mediates the release of mtDNA through translocation to mitochondria, thereby activating cGAS-STING pathway and aggravating renal IRI. Therefore, the mtDNA-cGAS-STING pathway is a key regulator of tubular inflammation that contributes to AKI and is a potential therapeutic target for preventing the progression of tubular inflammation-mediated kidney injury.

Inflammatory response is an important activation in maintaining body homeostasis. Involved in systemic immune response and inflammatory response, toll-like receptors (TLRs) can mediate immune cells to recognize pathogenic microorganisms and trigger systemic immune response (Jin et al., 2018). Toll like receptor 4 (TLR4) is an important pathogen pattern recognition receptor (Schattner, 2019), and it can initiate pro-inflammatory effects through various pathways including nucleotide binding oligomerization domain-like receptor protein 3 (NLRP3), myeloid differentiation factor 88 (MyD88), etc. (Wu et al., 2020; Ciesielska et al., 2021). When AKI occurs, the expression of TLR4 in kidney tissue is significantly increased, which triggers inflammatory response and leads to a series of pathological changes. TLR4 recognizes endotoxin and its downstream reaction play an critical role in the pathophysiology of AKI caused by lipopolysaccharide (LPS) (Mohamed et al., 2017). Studies have shown that the expression of TLR4 is significantly increased in animal models of LPS-induced AKI, and the increase of TLR4 level after LPS induction may be related to the increase of inflammatory cytokine mediated receptor expression. Epigallocatechin gallate (EGCG), the main catechin of green tea extract, is a powerful antioxidant and active oxygen scavenger (Eng et al., 2018). EGCG exhibits a protective effect against LPS-induced AKI by inhibiting the activation of TLR4/Myd88/NF-κB pathway.

The renal tissue of SAKI patients showed capillary endothelial injury, renal interstitial neutrophils and other inflammatory cell infiltration, and high expression of inflammatory factors, suggesting that inflammation plays an important regulatory role in the pathogenesis of SAKI (Zarbock et al., 2023). Lipoxin A4 (LXA4) has a significant negative regulatory effect on inflammation (Han et al., 2022), which can reduce sepsis-related inflammation and improve the survival rate of patients with sepsis (Hu et al., 2020). Activation of TLR4/Myd88/NF-κB pathway is one of the generally recognized mechanisms in inflammatory response. The activation of this pathway promotes the transcription of many pro-inflammatory cytokines and adhesion molecules such as IL-1B, IL-6, TNF-α, etc. These inflammatory mediators further activate the body’s defense system, resulting in continuous excessive release of inflammatory mediators, and ultimately lead to systemic inflammatory response syndrome characterized by the destruction of cells themselves (Feng et al., 2018). It is speculated that TLR4/Myd88/NF-κB signaling pathway aggravates renal tissue injury by mediating the production and release of inflammatory factors including IL-1B, IL-6 and TNF-α. Regulation of TLR4/Myd88/NF-κB signaling is of great significance in alleviating the occurrence and development of S-AKI.

Ferroptosis is involved in the development of AKI through pathological processes such as inflammation, endoplasmic reticulum stress and autophagy (Qiao et al., 2023). Zhu Z. et al. (2024) revealed that Ferroptosis inhibition Fer-1 and DFO promoted cell viability and reduced intracellular reactive oxygen species (ROS) production in contrast-induced AKI (CI-AKI). Besides, TMP significantly inhibited renal dysfunction, reduced AKI biomarkers, prevented ROS production, inhibited renal Fe^2+^ accumulation and increased glutathione peroxidase 4 (GPX4) expression. Regarding siRNA knockdown, plasmid overexpression of transferrin receptor (TFRC) and ferroptosis inhibitors, it indicates that TFRC-mediated ferroptosis plays a crucial role in CIN, whereas antioxidant TMP could exert an anti-ferroptosis effect to prevent such a pathological process by inhibiting TFRC and intracellular ROS production. Ferroptosis is also involved in various stages of IRI, and GPX4 and solute carrier family 7 members 11 (SLC7A11) are downregulated in renal tissue of IRI-induced AKI. Nuclear factor-E2-related factor 2 (NRF2), NADPH oxidase 1 (NOX1), and cyclooxygenase-2 (COX2) are upregulated, indicating the occurrence of Ferroptosis in IRI-AKI. Among them, non-coding microRNAs, miR-182-5p and miR-378a-3p target downregulate the expression of GPX4 and SLC7A11, which might induce the occurrence of Ferroptosis (Ding et al., 2020).

NRF2 plays a key role in regulating the occurrence and development of AKI by participating in ferroptosis (Hu et al., 2022). Studies (Zhang et al., 2021; Qiongyue et al., 2022) have shown that constitutionally activated NRF2 is closely related to the high incidence of various kidney diseases, and targeting NRF2 is regarded as an effective strategy for AKI treatment. NRF2 is an intracellular transcription factor that could protect against oxidative stress damage, and SLC7A11 is a substrate specific subunit of glutamate reverse transporter Xc-, and GPX4 is a core enzyme regulating the endogenous antioxidant system glutathione system. Therefore, NRF2 is a key factor in the classical pathway of ferroptosis. By down-regulating the expression of SLC7A11/GPX4, ferroptosis is induced, which interferes with the immune microenvironment of various diseases and affects the progression of diseases (Li P. et al., 2024; Yang et al., 2024). NRF2, as an upstream factor of the classical pathway of ferroptosis, could mediate the activation of the NRF2/SLC7A11/GPX4 axis to inhibit ferroptosis, interfere with the immune microenvironment of various diseases, and affect disease progression. Further, it is predicted that regulating the NRF2/SLC7A11/GPX4 axis-mediated process of ferroptosis and regulating the immune microenvironment to inhibit AKI progression might be the focus and direction of future research.

## 5 Conclusion

The bibliometric analysis offers a comprehensive overview of research trends and hotspots in the domain of immunisation associated with AKI. This method facilitates the visualisation of current research status and future trends. This study delved into the publication landscape of AKI-related immunisation research. Notably, the volume of publications is steadily increasing, with significant contributions from the United States and China. Furthermore, national institutions are providing substantial support for such research endeavours. Scholars exhibit unique research directions and demonstrate distinct preferences for particular journals. Keyword clustering analysis sheds light on current research hotspots, while the examination of highly cited and co-cited literature offers valuable guidance for newcoming peer studies. However, attention should be paied to insufficient collaborations among countries, institutions and researchers, which are significant for the future development of researches.

## 6 Limitations

While bibliometric analysis provides valuable insights into research focus and trends, it is not without limitations. First, the data source were solely from the WoSCC database, potentially introducing selective bias. Incorporating other databases such as PubMed and Scopus could enhance literature coverage and study accuracy. Second, as only English publications were retrieved, important articles in other languages may have been overlooked. Third, self-citation could introduce inherent bias in bibliometric analysis. Additionally, tools for bibliometric analysis, including CiteSpace and VOSviewer, may possess inherent limitations and biases that could impact analytic results. Finally, our search deadline was 10^th^ December 2023, and while the WoSCC database is updated daily, some significant studies may not have been included. Nonetheless, we believe this study has incorporated comprehensive publications up to 2023 and conclusions drawn remain robust even with the emergence of new data.

## Data Availability

The original contributions presented in the study are included in the article/Supplementary material, further inquiries can be directed to the corresponding authors.
